# SARS-CoV-19 Mutations: is a Blessing or a Curse for Human Being?

**DOI:** 10.4314/ejhs.v32i2.28

**Published:** 2022-03

**Authors:** Zohreh Jadali

**Affiliations:** 1 Department of Immunology, School of Public Health, Tehran University of Medical Sciences. Tehran, Iran

**TO THE EDITOR**: Adaptation to environmental change is principle of living things evolution and mutation is one of the main driving force of this natural process. Viruses are no exception and exploit different mechanisms for induction of mutations and genetic variation to ensure their survival. The rates of mutations varies among different types of viruses. For instance, RNA viruses have higher mutation rates than DNA viruses because of the error nature of viral RNA-dependent RNA polymerases(RdRp). The rate of new mutations may also be higher in some of the RNA viruses due to mutations in viral polymerase that can decrease polymerase fidelity. Although, high mutation rates are necessary for virus virulence and evolvability but almost they can compromise existing adaptations and can lead to viral extinction (1, 2). These observations have raised questions about the beneficial or deleterious effects of SARS-CoV-2 mutations.

At present, there is no clear cut answer for these questions. One important barrier is inadequate sequencing of SARS-CoV-2 variants for different reasons including, a lack of knowledge about mutations that occurred during the early stages of the outbreak or a limited number of sequencing studies regarding the roles of COVID-19 mutations on the virus evolution and biology. Moreover, some variants that cause less severe disease may be underrepresented in the databases because of their low frequency and eliminations. The genome of SARS-CoV-2 is a single-stranded RNA and has the following gene arrangement: 5′ untranslated region (UTR) [non-structural genes (ORF 1a/ORF1b replicase gene), structural genes [Spike(S), Membrane(M), Envelope(E), and Nucleocapsid(N)] and accessory genes (ORF3, ORF 6, ORF7a, ORF7b, ORF8, ORF9b)] 3′ UTR (3).

Available data indicates that mutations can act as a double-edged sword in COVID-19 infection. Some evidence suggests that genetic alterations can generate attenuated viruses. For instance, a deletion in the Nsp1-coding region (Δ500-532), has been reported for patients with COVID-19 that is correlated with lower viral load(lower infectiousness, lower transmissibility), decreased levels of IFN-β in serum and non-severe traits. Experiments have also shown that deletion of 382 nucleotides in the ORF8 region that is believed to have been involved in immune evasion could result in an attenuated SARS-CoV-2 phenotype (4).

These results are in line with those of several other studies that highlight the effects of deletions in ORF7, ORF8, and ORF10 on the reduction of SARS-CoV-2 virulance. D614G mutations on the important structural protein(S) that mediates coronavirus entry into host cells does not increase the disease severity. However, controversies surrounding this issue should not be avoided (5).

The harmful effects of COVID-19 genetic modifications on health constitute another important aspect that should be carefully considered. Because they can cause dramatic changes in functional properties of virus and may alter its infectivity, severity of disease and its interactions with host immune system. For instance, emerging evidence indicates that neutralization of some SARS-CoV-2 variants may be diminished by post-infection or postvaccination antibodies (6).

Although the exact mechanisms of these changes are unknown, advancements have been made recently. For instance, the E484K mutation that has been reported in S protein from different variants including Beta, Zeta, Eta and Iota can alters the electrostatic complementarity of antibody binding to the receptor binding domain(7). L18F is another mutation that occur in S protein of new variants(Beta, Gamma and Zeta) and negatively impacts the binding of neutralizing antibodies.

P71L mutation in the E protein is associated with disease severity and death rate. This mutation can also reduce the binding of SARS-CoV-2 to serum polyclonal neutralizing antibodies (8). Additionally, several mutations have been demonstrated in virus replication machinery enzymes, i.e., main protease (Mpro) and RdRp which may act through different mechanisms including reduction the hydrogen bonding potential or enhancement of the protein flexibility which is important for binding site recognition (6).

Structural analysis of SARS-CoV-2 nucleocapsid protein has also revealed another potential candidate gene for mutation discovery. These mutations have significantly different outcomes in vivo, as the mutation T205I deregulates N protein activation and interfere with the life cycle of virus. In contrast, the mutation S235F can alter the corresponding epitopes which in turn could be affecting the specificity of certain antibodies and change the vaccine-induced immunity against COVID-19 (6).

Despite these contradictory observations, it is important to consider that the survival and replication of the majority of viruses are completely rely on their host's cells. So, logically the inevitable necessity for a virus is to become less virulent and less lethal overtime. Fortunately, only a small percent of mutations can cause serious illness. Moreover, several lines of evidence indicate that the rate of virus evolution gradually reduces over time. An exonuclease activity with proofreading function in SARS-CoV-2 provide an explanation for reduced mutation rates. This ability allows the detection and correction of some errors that may arise during the process of copying genetic information, resulting in low-mutation-rate of virus. Moreover, functional and structural changes that induced by deleterious mutations can profoundly influence the reproduction of SARS-CoV-2. The accumulation of deleterious mutations that are detrimental to the virus along with the limited power of natural selection for weeding out harmful genetic mutations will gradually decrease the number of viruses and may result in mutational meltdown and reduction of viral evolutionary rates(9).

In summary, mutations could affect the pathogenesis of COVID-19 in many ways including altering the protein phenotype, disruption of virus structure or stability, and changing the binding affinity virus particles to cell surface receptors. So, it is important to know about the COVID-19 variants for transmission chain tracking, predicting the course of epidemics, development of effective vaccines and treatment strategies.
